# Limited similarity in microbial composition among coral reef fishes from the Great Barrier Reef, Australia

**DOI:** 10.1093/femsec/fiaf016

**Published:** 2025-02-06

**Authors:** Vincenzo A Costa, David R Bellwood, Jonathon C O Mifsud, Jemma L Geoghegan, Erin Harvey, Edward C Holmes

**Affiliations:** School of Medical Sciences, The University of Sydney, Sydney, NSW 2006, Australia; Research Hub for Coral Reef Ecosystem Functions, College of Science and Engineering, James Cook University, Townsville, QLD 4811, Australia; School of Medical Sciences, The University of Sydney, Sydney, NSW 2006, Australia; Department of Microbiology and Immunology, University of Otago, Dunedin 9016, New Zealand; Institute of Environmental Science and Research, Kenepuru, Porirua 5022, New Zealand; School of Medical Sciences, The University of Sydney, Sydney, NSW 2006, Australia; School of Medical Sciences, The University of Sydney, Sydney, NSW 2006, Australia

**Keywords:** cross-species transmission, Great Barrier Reef, metatranscriptomics, *Photobacterium damselae*, reef fish, virus

## Abstract

Reef fishes exhibit enormous biodiversity within a highly interactive ecosystem. Relatively little is known about the diversity and evolution of microbial species associated with reef fish, even though this may provide valuable insights into the factors that shape microbial communities. Through metatranscriptomic sequencing, we characterized the viruses, bacteria, and single-celled eukaryotes from 126 reef fish species inhabiting Lizard Island and Orpheus Island on the Great Barrier Reef, Australia. We assessed whether microbial communities differed between islands that are separated by 450 km, and to what extent fish viruses emerge in new hosts. Despite strong ecological interactions within the species-rich reef environment, and the presence of the same families of viruses on both islands, there was minimal evidence for the presence of individual viruses shared among fish species, reflecting low levels of cross-species transmission. Among bacteria, we identified the opportunistic bacterial pathogen *Photobacterium damselae* in apparently healthy cardinalfish species from both islands, indicating that these fish species are natural reservoirs. These data suggest that reef fishes have microbial–host associations that arose prior to the formation of the Great Barrier Reef, likely leading to strong host barriers to cross-species transmission and hence infectious disease emergence.

## Teaser

Metatranscriptomic sequencing reveals that the microbial species (viruses, bacterial, and eukaryotic parasites) present in fish that live at high density on the Great Barrier Reef, Australia rarely jump host species boundaries.

## Introduction

Symbiotic interactions are ubiquitous in nature and play an important role in animal, plant, and microbial evolution. In reef systems, the interdependence of corals, fishes, and their microbial symbionts supports fundamental ecological processes, and their dissociation may cause devastating declines in species abundance and reef functioning (Roth 2014). For example, the coral endosymbiont algae *Symbiodinium* provides a significant portion of the energy required for the survival of its host through photosynthesis (Roth 2014). In addition, oxidative stress—through anthropogenic stressors—can disrupt coral–algae symbiotic interactions and lead to coral bleaching with deleterious effects on reef system functioning, particularly for organisms that rely exclusively on coral for habitat and resources (Roth 2014). Healthy tropical coral reefs are characterized by remarkable biodiversity and exceptionally complex interactions. This provides an ideal forum for investigating the evolutionary and ecological factors shaping microbial communities, especially among teleost fishes. Teleost fishes are the most speciose group of vertebrates on coral reefs and rank among the most phylogenetically and ecologically diverse group of vertebrates, accounting for one-third of all currently described marine fishes (Spalding and Grenfell 1997, Eschmeyer et al. 2010). Reef fishes exhibit exceptional dispersal capabilities. Most have geographic ranges spanning thousands of kilometres, with some species covering approximately two-thirds of the global tropics (Hughes et al. 2002). This level of interconnectivity is also exhibited on individual reefs, characterized by highly complex food webs. Reef fishes display diverse trophic guilds (e.g. carnivores, mobile invertivores, omnivores, planktivores, sessile invertivores, and herbivores/detritivores), occur in a range of habitats (e.g. coral, sand, rubble, and caves), and commonly live in exceptionally close proximity (Bellwood and Wainwright 2002, Brandl et al. 2018, Siqueira et al. 2020).

This high degree of interaction is exemplified in the cryptobenthic reef fishes: behaviourally cryptic species with adult body sizes of ~5 cm or less that typically occupy the benthic zone (Brandl et al. 2018). Cryptobenthic reef fishes engage with larger reef fishes through extensive predator–prey interactions, and their frequent consumption is an integral component of coral reef food webs via the transfer of energy from microscopic prey to large predators (Mihalitsis et al. 2022). For example, the dwarf goby (*Eviota sigillata*) has a maximum lifespan of just 59 days and experiences mortality rates of 7.8% per day (Bellwood et al. 2006). In addition to predation, fishes interact through cleaning (including the removal of blood-sucking parasites) (Grutter 1999) and extensive food webs of coprophagy (consuming the faeces of other fishes) (Robertson 1982). The potential for microbial transmission on reefs is therefore considerable.

Although reef fish coexist in a highly diverse and interactive ecosystem, relatively little is known about whether and how microbiome composition (i.e. of viruses, bacteria, and eukaryotes) differs across fish groups within and among communities. Studies of microbial ecology in teleosts have largely focused on animals utilized in aquaculture and in laboratory model species, with very few investigations of wild ecosystems (Roeselers et al. 2011, Clements et al. 2014, Ghanbari et al. 2015). Fish–microbe interactions are highly beneficial for fish nutrition and immunity and are shaped by host and environmental factors including trophic level, age, water quality, and host phylogeny (Sullam et al. 2012, Bolnick et al. 2014, Clements et al. 2014, Wang et al. 2018, Geoghegan et al. 2021, Costa et al. 2023). For example, alterations in gut microbiome composition in response to external stimuli (such as starvation) can upregulate host immune genes (Ghanbari et al. 2015). In addition, fish microbiota play an important role in intestinal absorption and metabolism of fatty acids (Ghanbari et al. 2015).

Determining microbiome composition and the factors that shape host–microbe interactions in wild fish populations is imperative for identifying sources of risk to domestic fish populations. For example, metatranscriptomic studies have identified a wide spectrum of potential disease-causing agents in wild fish (Geoghegan et al. 2021, Costa et al. 2023) that may have the potential to be transmitted to farmed species through open net cages, coastal ponds, and wild sourced broodstock. As such, revealing wildlife reservoir hosts and the risk of microbial spillover at the domestic–wild interface will strengthen preparedness against emerging infectious diseases in aquaculture.

The extent of phylogenetic divergence between animal species has an important impact on virus ecology and evolution, particularly the likelihood of successful cross-species virus transmission, and hence is a key determinant of infectious disease emergence (Parrish et al. 2008, Longdon et al. 2011, Gupta et al. 2020, Shaw et al. 2020, French et al. 2023). The “phylogenetic distance” theory posits that microorganisms are more likely to be transmitted between closely related species that have conserved cellular properties, such as cell receptors (Longdon et al. 2014). This idea is supported by numerous studies across a broad spectrum of host taxa (e.g. vertebrates and invertebrates) and pathogen groups including virus–host interactions in reef fishes (Costa et al. 2023, French et al. 2023). For example, recent work has shown that reef fishes from a spatially restricted (100 m^2^) community from Orpheus Island in the Great Barrier Reef (GBR), Australia, harbour diverse viral assemblages that are highly host-specific despite ample opportunity for cross-species transmission (Costa et al. 2023).

With ~2500 coral reefs and 900 islands, fishes of the GBR constitute a natural model system to investigate spatial patterns of microbial evolution and diversity in vertebrate hosts. Molecular and fossil evidence indicates that the majority of reef fish families originated during the Paleocene and Eocene, ~66–50 million years ago (Ma). Subsequently, a notable acceleration in lineage diversification took place during the Oligocene and Miocene (34–5.3 Ma), with this shift occurring in the Indo-Australia Archipelago (IAA) (i.e. the IAA biodiversity hotspot) (Bellwood et al. 2017). During the Miocene (23–5.3 Ma), the reciprocal diversification of fish and coral species likely led to the development of the functional reef ecosystems that are observable today. By the early stages of the Pleistocene (5.3–0 Ma), almost all reef fish taxonomic groups were established and began their settlement on reefs across all tropical oceans (Bellwood et al. 2017), with the formation of the GBR fish communities likely occurring within the last 10 000 years following the stabilization of sea level to its current height ∼6000–8000 years ago (Bellwood and Wainwright 2002, Webster et al. 2018). Whether and how these colonization events shaped microbial evolution and diversity is unknown. For example, many reef fishes—particularly cryptobenthics that have limited dispersal—exhibit strong site fidelity, maintaining the same community composition year-round (Lefèvre et al. 2016). The seemingly consistent community composition might therefore lead to the generation of distinct microbial communities in different geographic areas, that will be most pronounced for rapidly evolving RNA viruses. Conversely, it is possible that the high ecological similarities among disjunct reef locations might result in broadly similar microbial compositions among fish communities.

We characterized the total assemblage of viruses, bacteria, and single-celled eukaryotes from 126 reef fish species spread across Lizard Island and Orpheus Island in the GBR. This study comprised 28 reef fish families, making it one of the largest investigations of microbial diversity and evolution in reef fish undertaken to date. Using metatranscriptomics, we examined whether viral and microbial communities differ between fish species from two islands separated by ∼450 km, and whether microorganisms are shared among fish species within each reef location. We also aimed to determine whether particular fish groups are potential reservoirs for important viral or bacterial pathogens. This is of particular importance given the high utilization of ornamental reef fish in aquaria, which face ruinous threats from emerging infectious diseases, as well as the ongoing threats of biodiversity loss on coral reefs from anthropogenic climate change, itself a significant contributor to the emergence of infectious diseases (Baker et al. 2022).

## Materials and methods

### Fish sample collection

Fish were collected under a GBR Marine Park Authority permit (G16/37684.1) and James Cook University Animal Ethics permit A2752. Building on transcriptome data from our previous sampling of reef fish from Orpheus Island on the GBR sampled in April 2021 (*n* = 192 fishes; 16 reef fish families) (Costa et al. 2023), an additional 163 individuals (24 families) were collected at Lizard Island (14° 40′ 08″ S 145° 27′ 34 ″E) during January 2022 (Table S3). All fishes were intact with no visible signs of disease, and the vast majority were adults. These animals were captured using an enclosed clove oil method (Costa et al. 2023) from both Mermaid Cove and the Lagoon entrance, located on the north and south side of Lizard Island. All fish caught were placed either dissected (liver and gills) or whole in RNAlater and then transported to the lab on ice. Specimens were stored at −80°C until RNA extraction. Overall, we sampled 1–12 individuals per species (with a mean of 3 per species).

### RNA extraction, metagenomic library preparation, and next-generation sequencing

As described previously (Costa et al. 2023) tissue specimens (e.g. liver and gills or whole fish) were collectively processed as a single extraction for each individual fish sample. The combined tissues were submerged in 600 µl of lysis buffer containing 30 µl of foaming reagent (Reagent DX; Qiagen, Hilden, Germany) and 60 µl of β-mercaptoethanol (Sigma-Aldrich). Tissue samples were homogenized with a TissueRuptor (Qiagen) for up to 1 min at 5000 rpm. The homogenate was centrifuged at maximum speed for 3 min to remove tissue residues. RNA was then extracted from the resulting clear supernatant using the RNeasy Plus Mini Kit (Qiagen), following the manufacturer’s guidelines.

RNA quantification was conducted utilizing a UV–Vis cuvette spectrophotometer (DeNovix, Delaware, USA) and a parallel capillary electrophoresis instrument (Fragment Analyzer; Agilent, CA, USA). RNA from individual fishes were pooled according to species, resulting in 79 RNA sequencing libraries newly generated from Lizard Island. All libraries were prepared using the TruSeq Total RNA Library Preparation Protocol (Illumina). Ribo-Zero Plus Kit (Illumina) was employed for host ribosomal RNA depletion, and paired-end sequencing (150 bp) was performed on the NovaSeq 6000 platform (Illumina). To mitigate index hopping and minimize false virus–host assignments, each library was sequenced on two different lanes. Library construction and metatranscriptomic sequencing were performed by the Australian Genome Research Facility.

### Assembly of reef fish viromes

We replicated the same methodology we used previously (Costa et al. 2023). Accordingly, raw RNA sequencing reads were quality trimmed using Trimmomatic v.0.38, employing the parameters SLIDINGWINDOW:4:5, LEADING:5, TRAILING:5, and MINLEN:25, and assembled into contigs using MEGAHIT (v.1.2.9), with default parameter settings (Bolger et al. 2014, Li et al. 2015). Assembled contigs were compared against the NCBI nonredundant protein (nr) and nucleotide (nt) databases (August 2022) using DIAMOND (BLASTX) (v.2.0.9) and BLASTn (Buchfink et al. 2015). To enable the identification of divergent viral sequences, we used an e-value search threshold of 1 × 10^−5^. Contigs with top matches to the kingdom “Viruses” (NCBI taxid: 10 239) were predicted as open reading frames (ORFs) using Geneious Prime (v.2022.0) (www.geneious.com) (Kearse et al. 2012). To remove false positives, all putative viral ORFs were translated into amino acid sequences and used as a query to perform a second search (BLASTP) against the NCBI nr database using Geneious Prime. ORFs with top matches to fish genes were deemed as false positives and removed from further analysis. To determine whether our putative viral contigs were expressed endogenous viral elements we screened for disrupted ORFs and flanking host regions using CheckV and BLASTn (Costa et al. 2023, 2024). Viral contig contamination and completion was determined using CheckV (Nayfach et al. 2021). Transcript abundances of both host (RPS13 gene) and virus (Costa et al. 2021, 2023, Geoghegan et al. 2021) were calculated using RNA-Seq by Expectation Maximization (v.1.3.0) and coverage was assessed by mapping using Bowtie2 (v.2.3.3.1) (Li and Dewey 2011, Langmead and Salzberg 2012).

### Taxonomic assignment and genome annotation of reef fish viruses

We aligned the amino acid sequences of our putative viruses (partial or complete) with the complete sequences of related viruses available on NCBI/GenBank using the E-INS-i algorithm in MAFFT (v.7.450) (Katon and Standley 2013). To determine whether our viruses were novel species, we used levels of sequence similarity and phylogenetic relationships (see below) as specified by the International Committee of Viral Taxonomy (ICTV) (https://talk.ictvonline.org) for each viral genus/family. Similarly, because vertebrate viruses are usually phylogenetically distinct from those that infect other host groups, we also used levels of sequence similarity and phylogenetic relationships to determine whether a virus was likely infecting reef fishes (i.e. vertebrate-associated) or of “non-vertebrate” origin, such as those derived from fish diet, microbiome, or environment (Costa et al. 2021, 2023, Geoghegan et al. 2021). Viral genomes were annotated with the Live Annotate and Predict tool in Geneious using reference sequences from NCBI/GenBank, with a similarity threshold of 20%. We also used the NCBI conserved domain (CDD) search tool and InterProScan with the TIGRFAMs (v.15.0), SFLD (v.4.0), PANTHER (v.15.0), SuperFamily (v.1.75), PROSITE (v.2022_01), CDD (v.3.18), Pfam (v.34.0), SMART (v.7.1), PRINTS (v.42.0), and CATH-Gene3D databases (v.4.3.0) (Jones et al. 2014).

### Viral phylogenetic analysis

To infer the evolutionary relationships of both the vertebrate and nonvertebrate associated viruses, we aligned the translated contigs with background protein sequences from each viral family/subfamily/genus selected from the ICTV classification and obtained from NCBI/GenBank. For RNA viruses, we used the conserved RNA-dependent RNA polymerase (RdRp), while for DNA viruses we used the DNA polymerase. Amino acid sequence alignments were trimmed using TrimAl (v.1.2) with a gap threshold of 0.9 and a variable conserve value (Capella-Gutiérrez et al. 2009). The best-fit model of amino acid substitution was estimated with the “ModelFinder Plus” (-m MFP) flag in IQ-TREE (v.1.6.12) (Nguyen et al. 2015, Kalyaanamoorthy et al. 2017). We used a maximum-likelihood approach to estimate phylogenetic trees using IQ-TREE, with 1000 bootstrap replicates. Trees were annotated using FigTree (v.1.4.4) (http://tree.bio.ed.ac.uk/software%20/figtree/).

### Virus nomenclature

Viruses were provisionally named (i.e. awaiting ICTV confirmation) according to host species (e.g. *H. melanurus* ranavirus) as described previously (Costa et al. 2023).

### Microbial profiling

To screen for transcripts associated with bacteria or single-celled eukaryotes, we aligned our contigs to a custom database comprising all nucleotide sequences available on NCBI (with the removal of environment or artificial sequences) using the KMA aligner and CCMetagen (Clausen et al. 2018, Marcelino et al. 2020). We also used this output to assess metazoan reads—e.g. arthropod, mollusc, platyhelminth, and nematode—that may represent potential vectors for virus transmission. For instances in which CCMetagen identified a microbe at the species level, we validated these taxonomic assignments by: (i) performing an additional search (BLASTn) against a custom 16S (bacteria) or 18S (eukaryote) rRNA database, and (ii) analyzing the BLASTX output (see above) for hits to bacterial or eukaryotic proteins. The contigs from these BLAST hits were predicted into ORFs, translated into amino acid sequences, and used as a query to perform a second search against the NCBI using BLASTP for further validation. The 16S or 18S rRNA gene was then utilized for phylogenetic analysis as a final validation. We focused our statistical analyses on bacteria more likely to be associated with fish tissues. For this reason, reads from cyanobacteria—that are consumed through fish diet—were removed from statistical analyses (Kavazos et al. 2022).

### Analysis of alpha and beta diversity

To compare viral and microbial communities between reef fish assemblages, we calculated beta diversity using a Bray–Curtis distance matrix with the phyloseq package in R (McMurdie and Holmes 2013). The variables assessed were host taxonomy, location (i.e. island) and trophic guild. Accordingly, fish species were categorized into six trophic guilds: carnivores, mobile invertivores, omnivores, planktivores, sessile invertivores, and herbivores/detritivores (Siqueira et al. 2020). We based our analysis on groups with three or more species. These data were then tested using permutational multivariate analysis of variance (PERMANOVA) with the vegan package (adonis) (Dixon 2003). All plots were constructed using ggplot2 in R (Valero-Mora 2010). We used measures of alpha diversity, which incorporates abundance and observed richness (i.e. the number of microbes) and beta diversity, to test for any differences in virome and microbiome composition between whole and dissected fish (Costa et al. 2023).

## Results

### Composition of sequence reads

**Figure 1.**
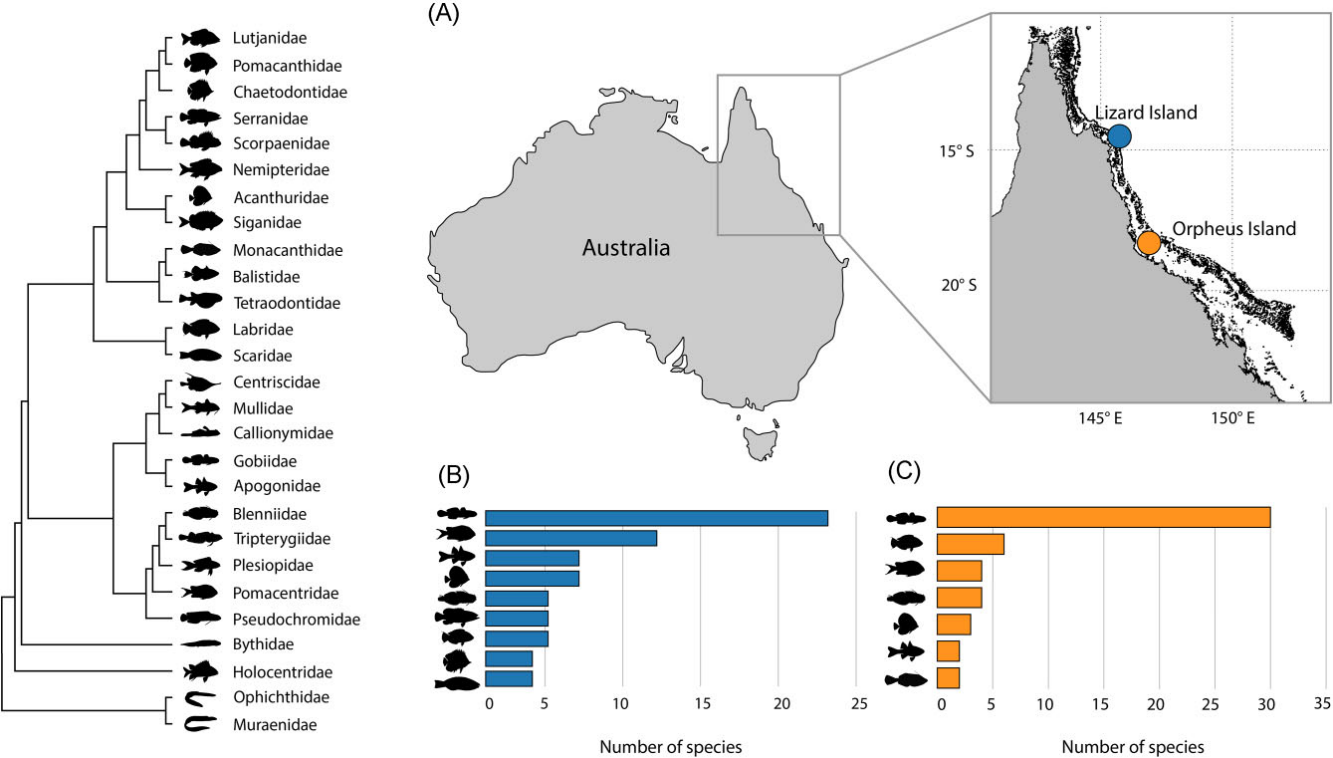
Sampling locations and taxonomic diversity of reef fish. (A) Location of Lizard Island and Orpheus Island in the GBR. (B) Taxonomy of samples collected from Lizard Island. (C) Taxonomy of samples collected from Orpheus Island. All other families with less than one species sampled are omitted from panels (B) and (C). Phylogeny obtained from Siqueira et al. (2020) and pruned to the reef fish families examined in this study.

We sequenced a total of 10.7 billion RNA reads, including 4.7 billion reads newly generated from Lizard Island fishes. Raw sequence reads have been deposited in the Sequence Read Archive (NCBI/SRA) under BioProject PRJNA1078998 and viral consensus sequences are available on GenBank under the accessions PP700469–PP700500. All Krona plots and tables are available on GitHub under the repository https://github.com/vcosta16/reeffishvirome. The remaining reads (Orpheus Island) are available on NCBI Sequence Read Archive (SRA) under BioProject PRJNA841039 (Costa et al. 2023). These data were generated from a total of 140 sequencing libraries, representing 126 reef fish species and 28 families (Fig. 1) (mean 54 617 146 reads per library). As expected, fish RNA accounted for 93% of the total reads, followed by RNA associated with cnidarians (5.7%), bacteria (0.31%), single-celled eukaryotes (0.29%), arthropods (0.23%), platyhelminths (0.18%), molluscs (0.04%), poriferans (0.03%), annelids (0.02%), nematodes (0.01%), fungi (0.009%), and viruses (0.009%) (Table S1).

To examine potential differences in virome and microbiome composition between whole and dissected fish (e.g. gills and liver), we used measures of alpha diversity which incorporates abundance and observed richness (i.e. the number of microbes) and beta diversity, which examines differences in microbial communities between samples. We identified no significant differences in viral abundance (*P* = .495) and observed viral richness (*χ*^2^ = 17.623, df = 1, *P* = .265) between whole and dissected fish. However, we identified significant differences in bacterial abundance (*P* = .013) and observed bacterial richness: whole fish contained a larger number of bacteria than dissected fish (*χ*^2^ = 25.064, df = 1, *P* = .004). We identified a higher abundance of microbial eukaryotic reads among dissected fish compared to whole fish (*P* = .006). However, we detected no significant difference in observed richness (*χ*^2^ = 9.4803, df = 1, *P* = .466). With respect to beta diversity, we found no clear partitioning according to sample type, with a high degree of overlap (Figs S1 and S2).

### Diversity and abundance of the reef fish virome

**Figure 2.**
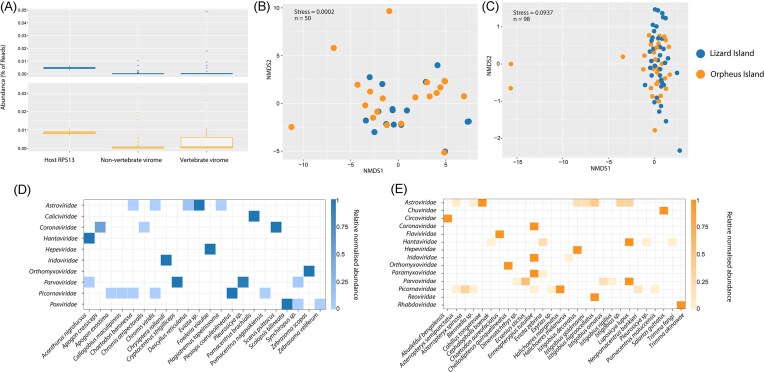
Diversity and abundance of the reef fish virome. (A) Abundance of host gene marker (RPS13), nonvertebrate virome, and vertebrate virome. (B) Nonmetric multidimensional scaling (NMDS) plot (Bray–Curtis dissimilarity matrix) for vertebrate-associated viruses for each fish species. (C) NMDS plot for nonvertebrate-associated viruses for each fish species. (D and E) Relative normalized abundance of vertebrate-associated viral families from fish species for each island.

We identified sequences representing 64 vertebrate-associated viruses (i.e. those likely infecting fish tissues), including 27 newly discovered from Lizard Island fishes (Table S2). The *Astroviridae* comprised 32.4% of vertebrate-associated viral reads, followed by the *Iridoviridae* (21.8%), *Picornaviridae* (18.6%), *Chuviridae* (15.9%), *Parvoviridae* (6.8%), and *Hantaviridae* (2.9%) with all other groups representing <1% of the total viral reads: the *Flaviviridae, Orthomyxoviridae (*order *Articulavirales), Poxviridae, Paramyxoviridae, Reoviridae, Circoviridae, Coronaviridae, Hepeviridae, Rhabdoviridae*, and *Caliciviridae* (Fig. 2). In the case of the Orpheus Island data, the presence of 36 of the 38 vertebrate viruses identified was previously confirmed using polymerase chain reaction (PCR) (Costa et al. 2023).

As well as vertebrate-associated viruses, we identified 194 viruses that were likely infecting porifera, arthropods, molluscs, fungi, plants, and microbial eukaryotes (e.g. dinoflagellates), including 100 that were newly discovered at Lizard Island. Because these viruses were likely associated with fish diet and not infecting the fish themselves, we assume that their presence does not reflect key aspects of fish biology (i.e. immunity or receptor binding) and hence effectively serve as a “negative control” in comparison to the vertebrate-associated viruses. We refer to this group as “nonvertebrate” viruses. The most abundant viral groups in this category were the *Flaviviridae* (24% of nonvertebrate viral reads), unclassified picornaviruses (i.e. “picorna-like” viruses) (22.5%), *Narnaviridae* (21.9%), *Nodaviridae* (8.9%), *Hepeviridae* (6.2%), *Partitiviridae* (4%), *Solemoviridae* (3.2%), *Negevirus* (2.1%), and *Totiviridae* (2%), with all other groups comprising <1%: *Reovirales, Weivirus, Qinviridae, Iflaviridae, Dicistroviridae, Rhabdoviridae, Picobirnaviridae, Quenyavirus, Bunyavirales, Chuviridae*, and *Tombusviridae*.

### Spatial comparisons of the reef fish virome

To determine whether viral composition (i.e. virus families or subfamilies) differed between Orpheus and Lizard islands, we analysed beta diversity using a permutational multivariate analysis of variance (PERMANOVA) with the Bray–Curtis dissimilarity matrix. This revealed no significant difference in vertebrate-associated viral communities on both islands (F = 1.42, *P* = .06), with overlapping viromes at the viral family/subfamily level (Fig. 2B). Accordingly, both islands contained viruses assigned to the *Picornaviridae, Astroviridae, Parvoviridae, Hantaviridae, Orthomyxoviridae, Coronaviridae, Hepeviridae*, and *Iridoviridae* (Fig. 2D and E). A similar pattern was observed when analysing the nonvertebrate virome (F = 1.53, *P* = .07) (Fig. 2C) with both islands dominated by unclassified picornaviruses, *Narnaviridae, Totiviridae, Partitiviridae*, and *Nodaviridae*.

### Evolutionary history and biogeographical patterns of vertebrate-associated viruses

While our analysis revealed similarities at the level of virus family/subfamily, almost all of the viruses identified exhibited levels of genetic divergence that reflected long-term virus–host associations, rather than recent cross-species transmission within each reef ecosystem. Indeed, the only instance of the same virus sequence shared between fish species was the presence of highly similar astroviruses (∼96 similarity across the entire genome) in gobies from Orpheus Island (Costa et al. 2023). No virus sequences were shared among the fishes sampled from Lizard Island.

We now describe the phylogenetic relationships of the shared viral groups in turn. We focus primarily on the relationships of viruses between both islands as well as those newly discovered at Lizard Island.

### Positive-sense single-stranded RNA viruses (+ssRNA): *Picornaviridae, Astroviridae, Hepeviridae, Caliciviridae*, and *Coronaviridae*

**Figure 3.**
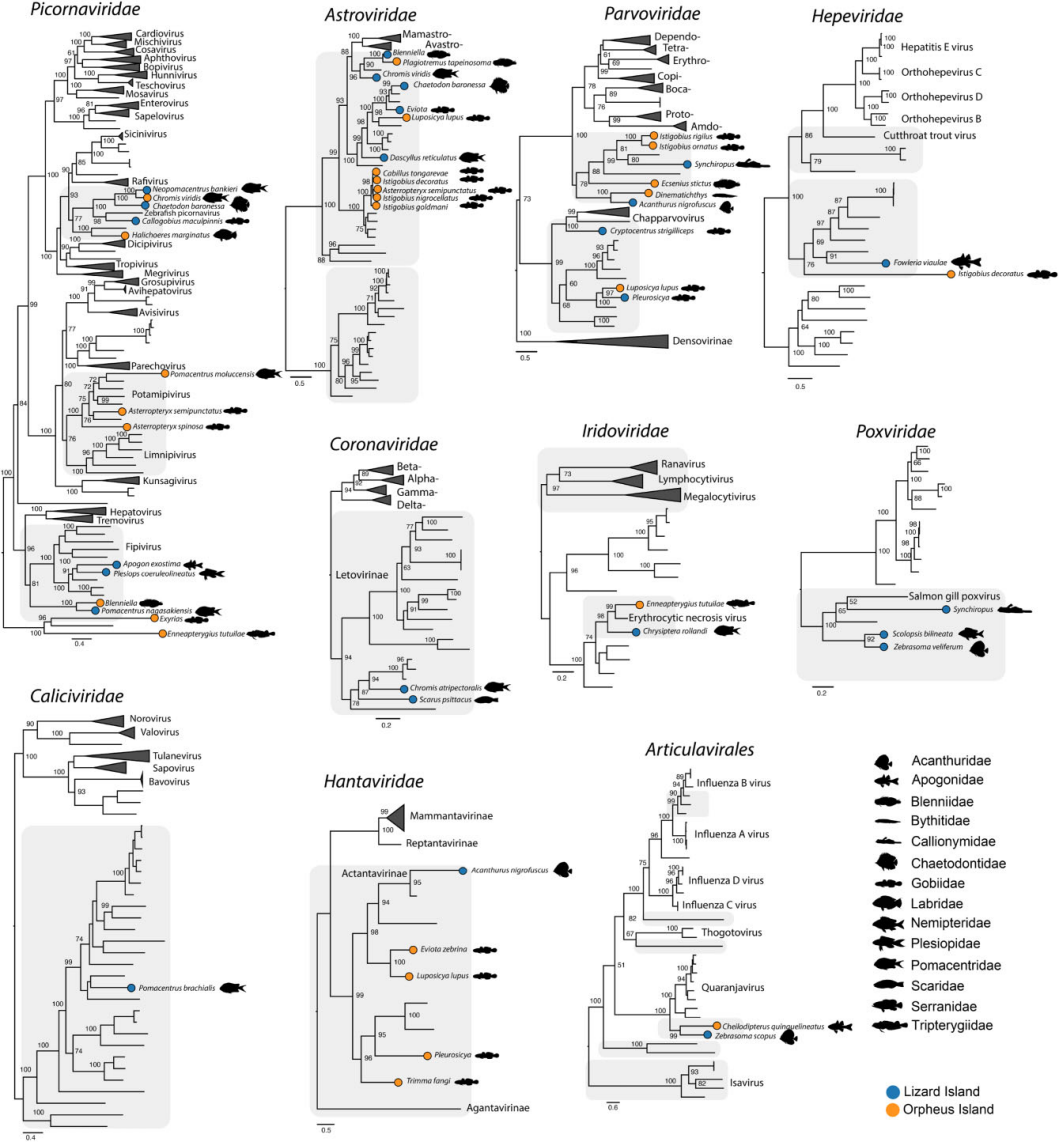
Phylogenetic relationships of reef fish viruses. Phylogenies were estimated using the RdRp gene for RNA viruses (*Picornaviridae, Astroviridae, Hepeviridae, Hantaviridae, Caliciviridae, Coronaviridae*, and *Articulavirales*), NS1 gene for parvoviruses and DNA polymerase for iridoviruses and poxviruses. Circles on branch tips represent viruses identified in this study. Fish silhouettes represent the fish family in which a virus was discovered. Scale bar represents the number of amino acid substitutions per site. Shaded branches represent fish viruses. Trees were midpoint rooted for clarity only.

The *Picornaviridae* were the most common viral group in our data set with 14 viruses: six from Lizard Island and eight from Orpheus Island. We discovered a group of four novel viruses that formed a distinct clade with the newly formed genus *Danipivirus*, represented by a single virus that is commonly detected in model zebrafishes (Altan et al. 2019) (Fig. 3). In this group, it was notable that we identified two relatively closely related viruses (70.6% sequence similarity across the entire polyprotein) in damselfishes (Pomacentridae) from both islands. A more distantly related virus was identified in *Chaetodon baronessa* (Chaetodontidae; 48%–49% RdRp similarity with both damselfish picornaviruses) suggesting that these viruses diversified within reef fish, although on an unknown time scale. Evidence of reef diversification was also identified in *Pomacentrus nagasakiensis* picornavirus and *Blenniella* picornavirus from Orpheus Island that exhibited 66.1% similarity and were related to fipiviruses found only at Lizard Island (Fig. 3).

We identified 12 astroviruses, including five novel viruses from Lizard Island. These were spread across three major clades: clade I, represented exclusively by fish viruses including five goby viruses from Orpheus Island (see above); clade II, similarly represented by fish viruses including those from both Lizard and Orpheus Island; and clade III that fell sister to the genera *Mamastrovirus*, found in mammals and *Avastrovirus*, exclusively in birds (Fig. 3). Notably, both *Plagiotremus tapeinosoma* astrovirus (Lizard Island) and *Blenniella* astrovirus (Orpheus Island) fell within clade III, exhibiting 75.8% sequence similarity in the RdRp gene. Similarly, both *Eviota* astrovirus and *C. baronessa* astrovirus clustered together within clade II, while the other reef fish viruses were more divergent.

Among other +ssRNA viruses, we identified two hepeviruses—*Istigobius decoratus* hepevirus (Orpheus Island) and *Fowleria viaulae* hepevirus (Lizard Island)—that grouped with other fish hepeviruses in phylogenetic trees, as well as a novel calicivirus in *Pomacentrus brachialis* that similarly fell within a broad group of fish and amphibian caliciviruses (Fig. 3).

Both letoviruses (*Coronaviridae*, subfamily *Letovirinae*)—*Chromis atripectoralis* letovirus and *Scarus psittacus* letovirus—from Lizard Island formed a basal clade with *Microhyla letovirus* and three other viruses identified in African cichlids (Miller et al. 2021, Costa et al. 2024). Together, this group likely formed a novel genus within the *Letovirinae* that includes diverse ectothermic hosts such as amphibians, jawless and ray-finned fishes (Miller et al. 2021).

### Negative-sense single-stranded RNA viruses (-ssRNA): *Hantaviridae* and *Articulavirales*

A notable observation from our previous sampling of reef fish was the detection of hantaviruses in four different goby species at Orpheus Island. In contrast, at Lizard Island we only detected one hantavirus in a surgeonfish (*Acanthurus nigrofuscus*) that was sister to Wenling red spikefish hantavirus (NCBI/GenBank accession number AVM87662.1) and was highly divergent to those found at Orpheus Island (∼29% RdRp similarity). Overall, these viruses fell within the subfamily *Actantavirinae*, that exclusively infects ray-finned fishes. Similarly, we detected two viruses with high levels of divergence (40% similarity) from both islands that were related to quaranjaviruses (order *Articulavirales*) (Fig. 3).

### DNA viruses

We identified viruses from three lineages within the *Parvoviridae*: the *Parvovirinae, Chapparvovirus*, and *Ichthamaparvovirus* groups. Reef fish *Parvovirinae* fell as a sister lineage to mammalian and avian viruses (e.g. *Dependoparvovirus* and *Aveparvovirus*), again with high sequence divergence between islands. For example, the closest relatives among both islands were *A. nigrofuscus* parvovirus (Lizard Island) and *Dinematichthys* parvovirus (Orpheus Island) that shared 62% NS1 gene sequence similarity (Fig. 3). Notably, we identified a novel chapparvovirus in *Cryptocentrus strigilliceps* that was related to tilapia parvovirus—a pathogen in farmed Tilapia in China—making it the second fish virus identified in this group (Liu et al. 2020) Among the genus *Ichthamaparovirus*, both *Pleurosicya* icthamaparvovirus (Lizard Island) and *Luposicya lupus* ichthamaparvovirus (Orpheus Island) shared a common ancestor (57.9% NSI similarity) and formed a distinct clade with other fish-infecting parvoviruses (Pénzes et al. 2019).

Similar patterns were observed in reef fish *Betairidovirinae* (*Iridoviridae*), between both *Chrysiptera rollandi* iridovirus (Lizard Island) and *Enneapterygius tutuilae* iridovirus (Orpheus Island). These viruses exhibited 82.2% similarity in the conserved major capsid protein (MCP) and clustered with erythrocytic necrosis virus (ENV).

Poxviruses were only identified at Lizard Island, with phylogenetic analysis placing them with other fish viruses—Salmon gill poxvirus and carp edema virus—within the subfamily *Chordopoxvirinae*. Notably, *Zebrasoma veliferum* poxvirus and *Scolopsis bilineata* poxvirus shared a common ancestor (80.3% similarity), strongly suggestive of an origin in reef fish (Fig. 3).

### Close phylogenetic relationships of nonvertebrate-associated viruses

**Figure 4.**
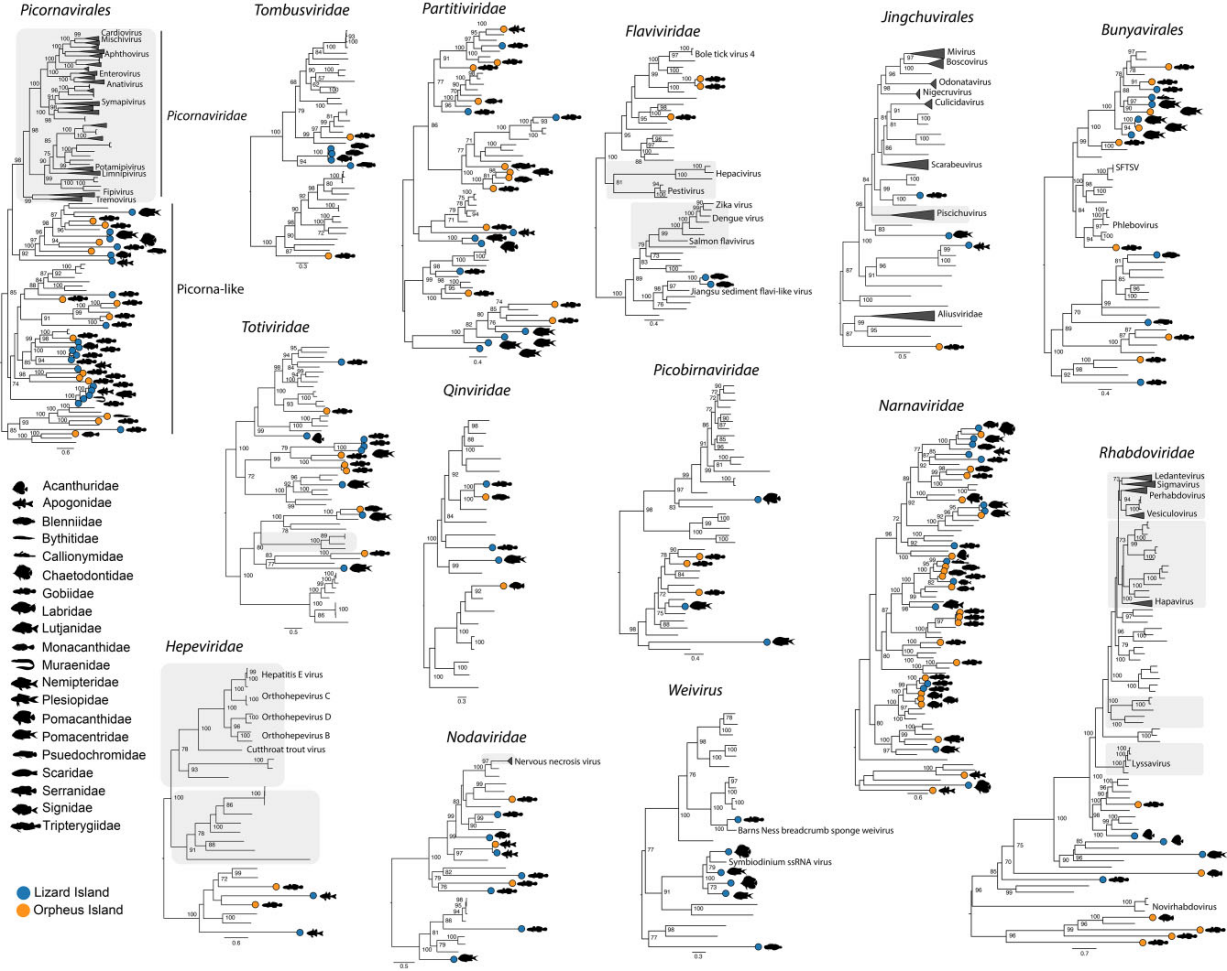
Close phylogenetic relationships among nonvertebrate-associated viruses. Phylogenies were estimated using amino acid sequences of the RdRp gene. Circles on branch tips represent viruses identified in this study. Fish silhouettes represent the fish family in which a virus was discovered. Scale bar represents the number of amino acid substitutions per site. Shaded branches represent vertebrate-associated viruses. Trees were midpoint rooted for clarity only.

A common observation in our data set was that genetically diverse reef fish exhibited very similar assemblages of nonvertebrate-associated viruses (Fig. 2C). This pattern sits in marked contrast to the vertebrate-associated viruses, that were rarely shared among species. In particular, we identified the same virus (i.e. 98%–99% similarity) in multiple host species within the following groups: unclassified picornaviruses, *Tombusviridae, Totiviridae, Nodaviridae, Narnaviridae*, and *Bunyavirales* (Fig. 4). As expected, most of these cases of virus sharing occurred within each island. For example, we identified the same tombusvirus in two blennies and one wrasse from Lizard Island as well as the same narnavirus in three gobies from Orpheus Island (Fig. 4). A notable exception was the presence of two nodaviruses identified in *Fowleria vaiulae* from both islands that exhibited 98% similarity in the RdRp gene. While nodaviruses are capable of infecting fish species (e.g. nervous necrosis virus), we detected reads associated with decapods in both libraries. These reads, in combination with the phylogenetic positions of these viruses (i.e. highly divergent from nervous necrosis virus and more related to crustacean viruses) strongly implies that these viruses are of dietary origin.

### Diversity and abundance of bacteria in reef fish assemblages

**Figure 5.**
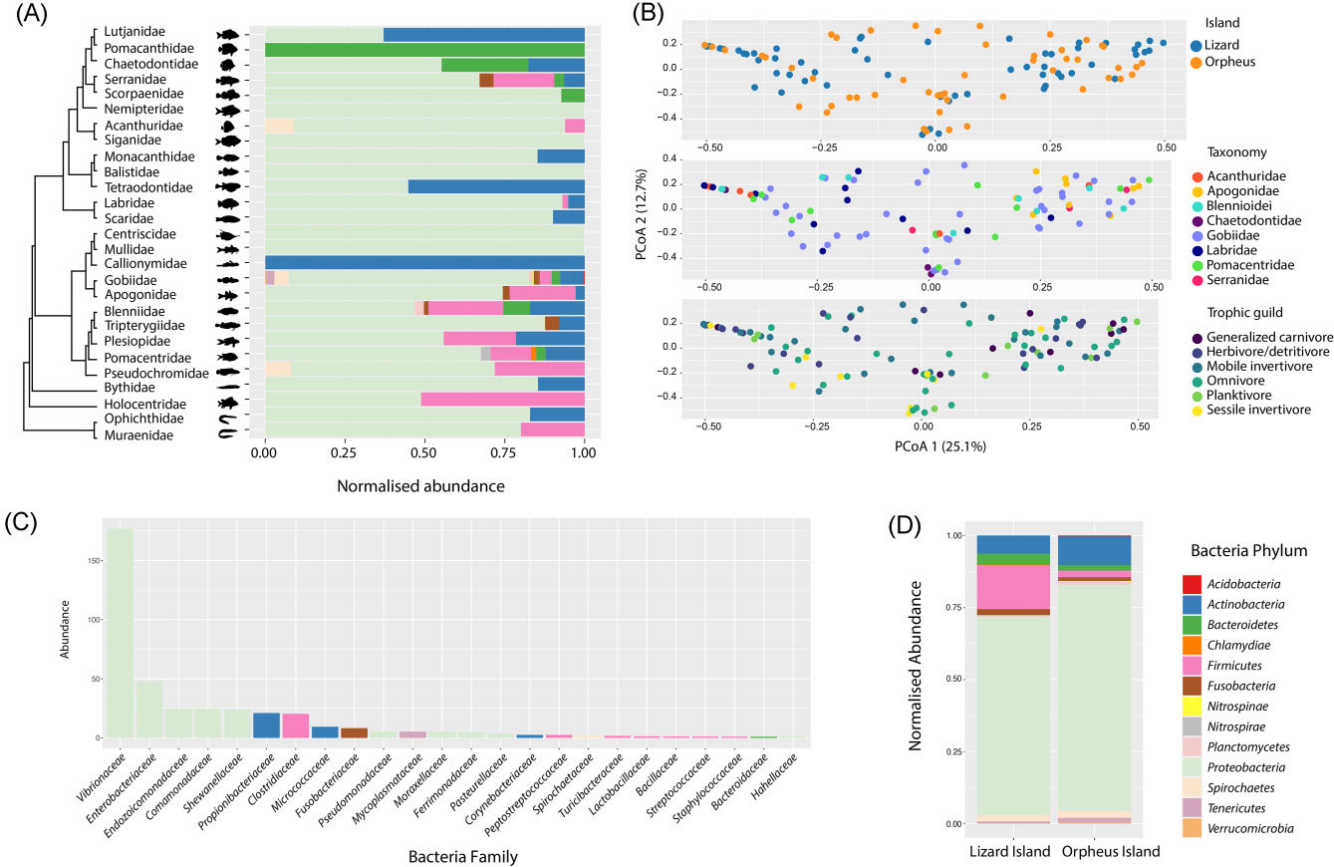
Composition of bacteria in reef fish. (A) Normalized abundance of bacterial phyla for each reef fish family. (B) Principal coordinate analysis (PCoA) plots of bacterial communities for island, host taxonomy, and trophic guild using Bray–Curtis dissimilarity matrix. (C) Abundance of bacterial families coloured by phylum. (D) Normalized abundance of bacterial phyla for each island based on microbial profiles of each fish species examined in this study.

After the removal of cyanobacteria—that are consumed through fish diet—the phylum *Proteobacteria* accounted for 73.5% of the total sequence reads from bacteria, followed by *Firmicutes* (9%), *Actinobacteria* (8%), *Bacteroidetes* (2.9%), *Spirochaetes* (2.3%), and *Fusobacteria* (1.7%), with all other phyla each representing <1% (Fig. 5A). At the family level, *Vibrionaceae* (42.7%) and *Enterobacteriaceae* (11.6%) were present at the highest frequencies followed by *Endozoicomonadaceae* (5.9%), *Comamonadaceae* (5.9%), *Shewanellaceae* (5.8%), *Propionibacteriaceae* (5%), *Clostridiaceae* (4.9%), *Micrococcaceae* (2.3%), *Fusobacteriaceae* (2%), *Pseudomonadaceae* (1.3%), and *Mycoplasmataceae* (1.3%) (Fig. 5).

To examine whether host phylogeny, biography, and ecology impacted bacterial diversity, we performed a principal coordinate analysis (PCoA). This revealed no partitioning according to reef location with largely overlapping bacterial communities between the two islands (*F* = 1.711, *R*^2^ = 0.015, *P* = .082) (Fig. 5). PERMANOVA revealed significant differences in bacterial composition between fish taxonomic groups (*F* = 2.42, *R*^2^ = 0.153, *P* = .001). However, there was overlap among all fish species, particularly the gobies, which may be explained by their high involvement in coral reef food webs (Bellwood and Wainwright 2002, Brandl et al. 2018) (Fig. 5B).

To assess the possible impact of fish ecology on bacterial composition, we grouped fish species into six trophic guilds: carnivores, mobile invertivores, omnivores, planktivores, sessile invertivores, and herbivores/detritivores (Siqueira et al. 2020). While we detected significant differences in bacterial composition between these groups (*F* = 1.485, *R*^2^ = 0.074, *P* = .035), there was similarly high overlap with no clear partitioning according to host trophic guild (Fig. 5B).

**Figure 6.**
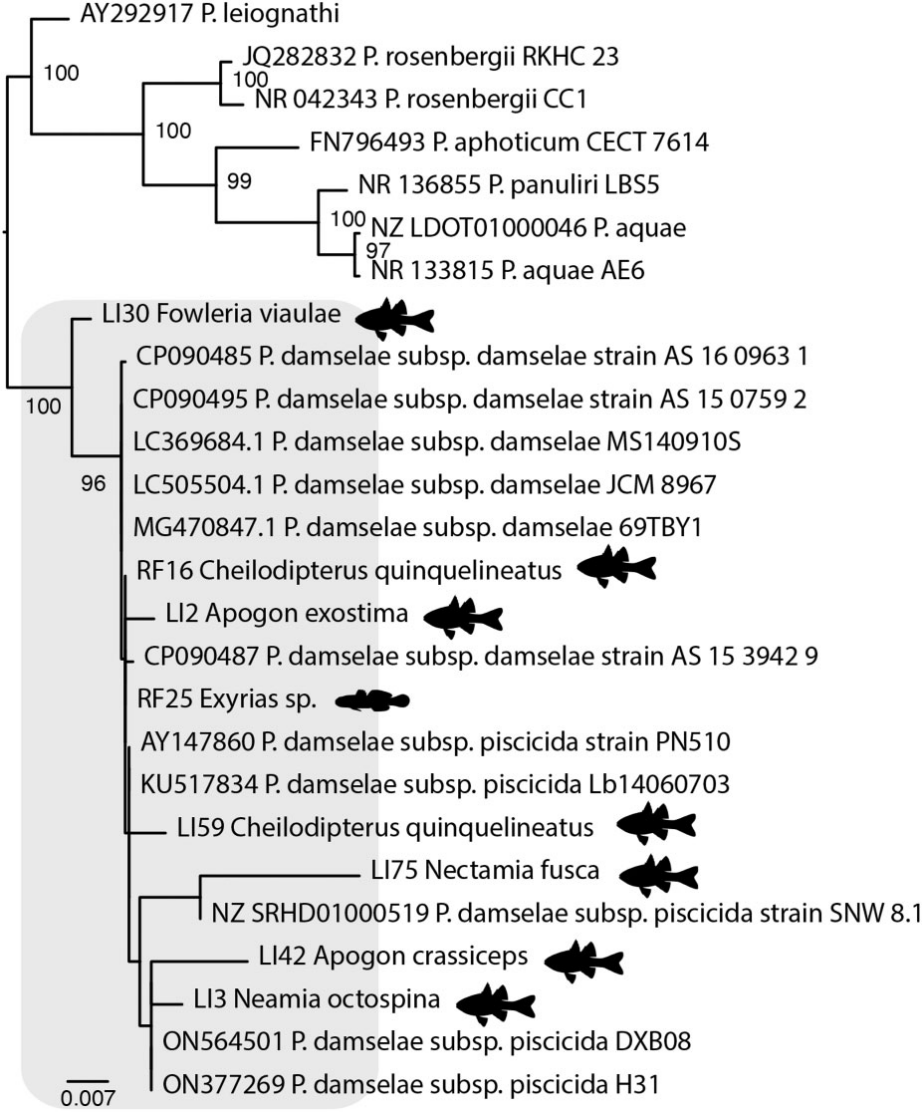
Phylogeny of the genus *Photobacterium*. The maximum-likelihood phylogeny was estimated using nucleotide sequences of the 16S gene. Fish silhouettes represent individuals identified in this study. The scale bar represents the number of nucleotide substitutions per site. Tree was midpoint rooted for clarity only.

It was notable that we identified *Photobacterium damselae* in 88% of the cardinalfish species examined from both islands (Fig. 6). We also detected other *Vibrionaceae*, including four that were related to those within the *Vibrio harveyi* clade: *V. parahaemolyticus, V. campbellii*, and *V. owensii*. (Fig. S3). In addition, we identified *V. fortis*—an opportunistic pathogen of coral—in the surgeonfish, *Ctenochaetus binotatus* (Sun et al. 2023) (Fig. S3).

### Composition of single-celled eukaryotes

Finally, we identified transcripts representing single-celled eukaryotes from 15 phyla. Dinoflagellates were the most abundant group (46% of the total eukaryotic reads) followed by Bacillariophyta (21.6%), Foraminifera (12.6%), Cercozoa (5.8%), Euglenozoa (2.9%), Apicomplexa (2.7%), Ciliophora (2.5%), Endomyxa (1.1%), Parabasalia, Haptista, Fornicata, and Heterolobosea (all <1%). Fungi—Ascomycota, Basidiomycota, and Microsporidia—were identified at much lower frequencies, representing only 3.3% of the total reads in this category.

**Figure 7.**
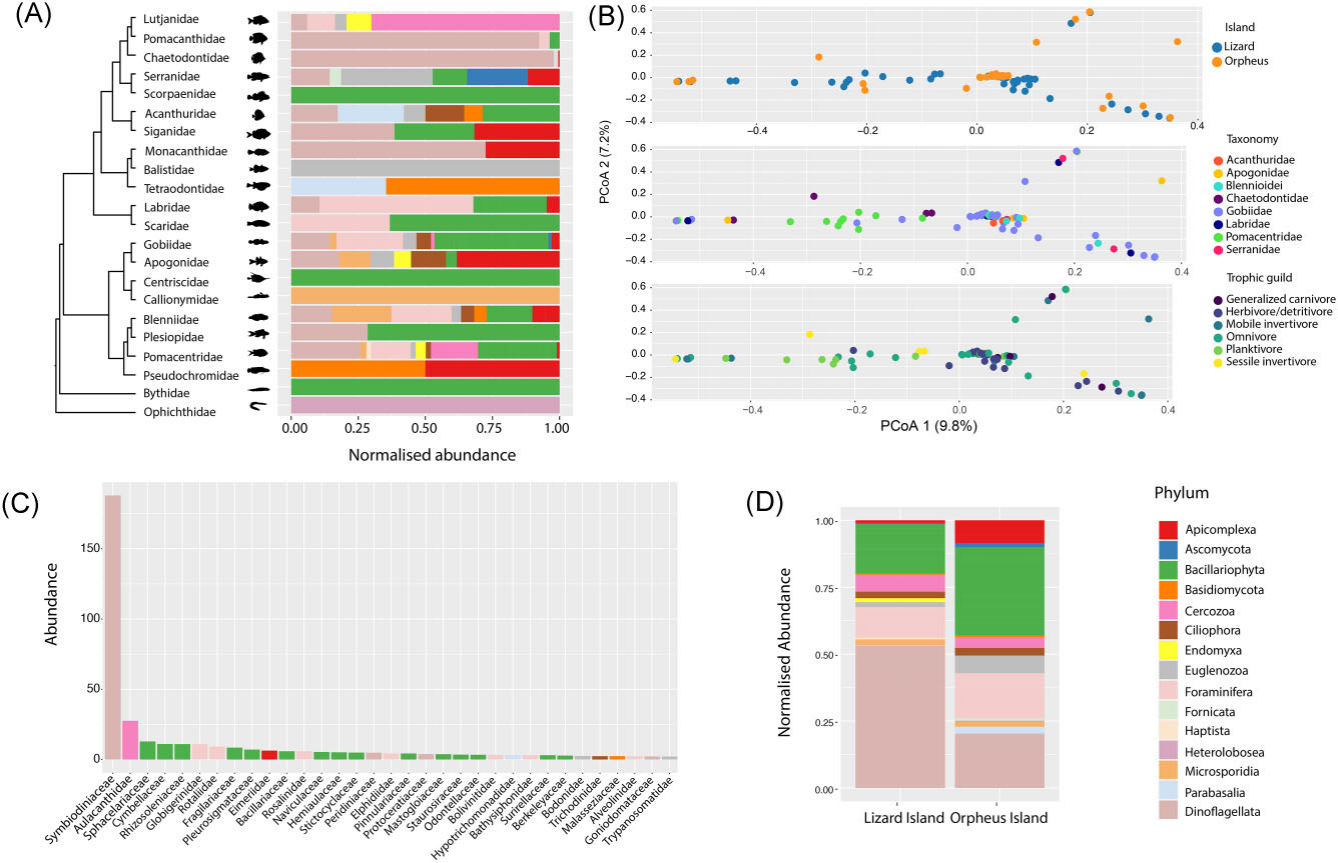
Composition of eukaryotes in reef fish. (A) Normalized abundance of single-celled eukaryotic phyla for each reef fish family. (B) PCoA plots of single-celled eukaryotic communities for island, host taxonomy, and trophic guild using Bray–Curtis dissimilarity matrix. (C) Abundance of single-celled eukaryotic families coloured by phylum. (D) Normalized abundance of single-celled eukaryotic phyla for each island based on microbial profiles of each fish species examined in this study.

Using these data, we performed a PCoA based on fish location, taxonomy and ecology. As with the analysis of virome composition, this revealed overlapping microbial communities between both islands (*F* = 1.208, *R*^2^ = 0.015, *P* = .181). However, there were significant differences in microbial communities between fish taxonomic groups (*F* = 1.384, *R*^2^ = 0.119, *P* = .002), which may be driven by the separation of pomacentrids from other groups, in turn reflecting the larger diversity of microorganisms identified in this group (Fig. 7A and B). When assessing the impact of host ecology, we similarly identified substantial overlap with no clear partitioning (Fig. 7). Among the Apicomplexa, which are common parasites of vertebrates, we detected reads associated with *Goussia* spp. in *Chaetodon aureofasciatus, Halichoeres melanurus*, and *Eviota melasma*. Overall, the parasitic families, such as the Eimeriidae (Apicomplexa), Trypanosomatidae, and Ichthyobodonidae (both Euglenzoa) were found in eight libraries at low transcript abundances, representing an average of 0.19% of the reads in each library.

## Discussion

Our metatranscriptomic analysis of 126 reef fish species revealed no significant differences in the composition of viral and microbial families/subfamilies between two islands located ∼450 km apart in the Australian GBR. Fish sampled from islands were associated with the presence of the *Picornaviridae, Astroviridae, Parvoviridae, Hantaviridae, Orthomyxoviridae, Coronaviridae, Hepeviridae*, and *Iridoviridae*, as well as *Proteobacteria* of the *Vibrionaceae, Enterobacteriaceae*, and *Endozoicomonadaceae*. Also of note was that we observed high levels of genetic diversity within each group of viruses, reflecting low levels of virus sharing between fish species (both within and between islands), despite strong ecological interactions in the reef ecosystem and our spatially restricted sampling. These data suggest that the long-term evolutionary history of reef fishes, in line with the ecological similarities between both islands, have resulted in broadly similar virome compositions with strong host specificity. From an evolutionary perspective, the consistent community compositions within reef environments (Lefèvre et al. 2016), its ecological stability (i.e. lack of seasonal variation), combined with phylogenetic divergence between host species, likely favours strong host specialization.

These findings offer further support for recent studies of virus ecology in fish that show that host phylogeny has a strong influence on virome composition: viruses are more likely to jump between closely related host species that exhibit similar cellular components (Costa et al. 2023, 2024). For example, closely related African cichlid species, that have evolved *in situ* within Lake Tanganyika over the last 10 million years, exhibit highly similar viromes with high levels of cross-species transmission and viral generalism, likely due to reduced host barriers to infection compared to reef fishes (Costa et al. 2024). African cichlids are members of a single family, the Cichlidae, and exhibit some of the lowest pairwise genetic distances observed between vertebrates (e.g. differences of 0.03% between some species). In contrast, reef fish communities are composed of several divergent families, including 28 examined in this study (Siqueira et al. 2020, Svardal et al. 2021), many of which were established around 66 Ma, occupying the biogeographic region—Tethys—now covered by Europe and the Mediterranean Sea (Bellwood et al. 2017). During the Oligocene there was a shift in species richness from Tethys to the IAA, eventually forming the IAA biodiversity hotspot (Cowman and Bellwood 2013, Bellwood et al. 2017). Most reef fish genera formed within the IAA around 30–15 Ma, with accelerated speciation during the Miocene (Bellwood et al. 2017)

A notable difference between cichlids and reef fishes, which may explain their strikingly contrasting levels of cross-species transmission, is that cichlids rapidly evolved within Lake Tanganyika, while reef fishes diversified within the IAA, over many millions of years, prior to their settlement on the GBR (Bellwood et al. 2017, Ronco et al. 2021). As the GBR that exists today formed after Pleistocene sea level rises, reef fish communities only became established during the last ∼10 000 years, such that the genetic boundaries inhibiting cross-species transmission were already established in the IAA before their settlement at reef locations. In marked contrast, the adaptive radiation of the African cichlids would have provided a more favourable environment for cross-species transmission as their diversification occurred rapidly within the confinements of Lake Tanganyika, generating a large pool of closely related host species for infection. Indeed, a time-calibrated phylogeny of cichlid hepaciviruses showed that elevated rates of virus diversification coincided with a period of rapid cichlid speciation (Costa et al. 2024).

We identified a high prevalence of the opportunistic bacterial pathogen *P. damselae* among cardinalfishes (Apogonidae), further illustrating a phylogenetic effect. However, it is likely that this association represents a long-term symbiotic relationship between cardinalfishes and *P. damselae*, particularly as it was detected in *Cheilodipterus quinquelineatus* from both islands, as well as its presence in 88% of the cardinalfish libraries examined. This strongly suggests that *P. damselae* forms part of the natural microbiome in these species. It is noteworthy that most cardinalfish species are nocturnal on coral reefs, and the genus *Photobacterium* is renowned for its bioluminescence—via the expression of *lux* genes—including some strains of *P. damselae* (Urbanczyk et al. 2011). Indeed, *Photobacterium mandapamensis* is a bioluminescent symbiont of the urchin cardinalfish, *Siphamia tubifer*, where it provides light to attract prey (Urbanczyk et al. 2011, Gould et al. 2022). Several cardinalfish species have evolved specialized “light organs” that harbour bioluminescent *Photobacterium*; however, these specialized organs are not present in all species (Thacker and Roje 2009). It is important to note that we did not observe the expression of *lux* genes in any of our libraries, and these species are not recognized for possessing a bioluminescent system. The presence of *P. damselae* in almost all Apogonidae libraries implies a longstanding relationship between *Photobacterium* and cardinalfishes on coral reefs, suggesting that this genus might have originated and diversified in the reef environment.

*Photobacterium damselae* is an opportunistic pathogen in aquaculture across Africa, Asia, Europe, and Australia, affecting a wide range of marine fishes, including barramundi (*Lates calcarifer*) (Gouife et al. 2022). Free-living *P. damselae* can persist in seawater for prolonged periods and may opportunistically infect a new host through skin lesions (Gouife et al. 2022). That we primarily detected this bacterium in cardinalfishes among 27 other reef fish families further suggests that these species serve as natural hosts for *P. damselae* and should be monitored closely, particularly if interacting with farmed populations or used commercially in the aquarium trade (Gouife et al. 2022). For example, following marine prawn farming, barramundi farming is the second largest aquaculture activity in Queensland, where the GBR is located (www.business.qld.gov.au). Similarly, wild ornamental fishes are routinely collected on a commercial basis on the GBR (Whitehead et al. 1986). Ornamental fishes, such as those utilized in the aquarium trade, are known to carry and spread microorganisms with often devastating impacts in aquaculture. A case in point was the emergence of infectious spleen and kidney necrosis virus in farmed Murray cod—an iconic fish species in Australia—that was linked to the importation of ornamental fishes (Go and Whittington 2006).

The identification of two iridoviruses that were related to ENV (83%–89% MCP similarity) was also noteworthy. ENV is an important pathogen in the North Atlantic and North Pacific oceans (Pagowski et al. 2019), and the presence of an ENV-like virus at Lizard Island is consistent with a previous study that identified viral erythrocytic necrosis in a juvenile triggerfish (*Rhinecanthus aculeatus*) at this location (Davies et al. 2009). Moreover, this study identified VEN-like bodies in *R. aculeatus* erythrocytes that were also found in the digestive tract of associated gnathiid isopods that are common blood feeding parasites. Similarly, we detected reads from gnathiids in *C. rollandi* from Lizard Island, implying that gnathiids could act as vectors in the marine environment (Davies et al. 2009), although we did not detect any gnathiid reads in *E. tutuilae* from Orpheus Island. This association warrants further investigation and has the potential to improve control measures against pathogenic ENV in susceptible species such as pink (*Oncorhynchus gorbuscha*) and chum (*Oncorhynchus keta*) salmon (Pagowski et al. 2019).

It was notable that we identified a low number of protozoan parasites (*n* = 8), such as apicomplexans and trypanosomes. In a similar manner, we detected low levels of fungal reads (e.g. Ascomycota, Basidiomycota, and Microsporidia), supporting the idea that the marine environment, particularly coral reefs, contains low fungal biomass perhaps because of the oligotrophic conditions compared to nutrient-rich terrestrial environments (Roik et al. 2022). The vast majority of reads in this category were associated with dinoflagellates, diatoms, and foraminifera that likely came from the reef environment, particularly dinoflagellates that form symbioses with corals (Roth 2014). Indeed, we discovered six viruses that fell within the “Weivirus” group—a group of dinoflagellate, poriferan, and mollusc viruses—grouping with a virus recently identified in *Symbiodinium* s. (Shi et al. 2016, Benites et al. 2022) (Fig. 4).

While our microbial profiling was able to detect diverse microbial communities, it is important to note that our sampling was initially performed for virological analysis (Costa et al. 2023), such that our methodology was optimized for the detection of viruses (e.g. the depletion of ribosomal RNA). This may have limited our ability to detect bacteria and microbial eukaryotes. Moreover, there were necessary limitations in our sampling that impacted the power of our statistical analyses. For example, there was a necessarily higher number of gobies sampled compared to other reef fish families: these numbers reflect the high diversity and abundance of these species on coral reefs (Brandl et al. 2018). In addition, these comparisons were based on combined tissues, such as liver and gills or whole fish (i.e. cryptobenthic reef fishes), which may have affected microbial discovery and statistical analyses of microbial ecology. Finally, as metatranscriptomics is based on RNA-sequencing, it is necessarily the case that we were only able to detect microbes and DNA viruses that were expressing genes at the time of sampling. As a result, metatranscriptomics may be less effective for microbial profiling compared to DNA-based methods such as 16S/18S sequencing. Finally, it is important to note that our approach does not directly assess transmission pathways, limiting its ability to inform on the mechanisms by which viruses jump between host species. Therefore, these results should be interpreted with caution, as they represent taxonomic associations rather than definitive evidence of cross-species transmission.

In summary, while our metatranscriptomic analysis of reef fish communities from islands separated by ∼450 km revealed the same viral and bacterial families across both islands, there was strikingly little evidence for the presence of viral and microbial species shared between reef fishes. As such, these data support the concept that fish are rich in microbial diversity, but that there are strong barriers to infection in host communities that display high levels of genetic diversity.

## Supplementary Material

fiaf016_Supplemental_Files

## Data Availability

Raw sequence reads have been deposited in the Sequence Read Archive (NCBI/SRA) under BioProject PRJNA1078998. All viral sequences discovered have been deposited in NCBI/GenBank under the accessions PP700469–PP700500. All phylogenetic trees, tables, and Krona plots are available on GitHub under the repository https://github.com/vcosta16/reeffishvirome.
