# Supplementary material for: Limited similarity in microbial composition among coral reef fishes from the Great Barrier Reef, Australia
Source: FEMS Microbiol Ecol. 2025 Feb 6;101(3):fiaf016. doi: 10.1093/femsec/fiaf016 (PMC11879539; doi:10.1093/femsec/fiaf016)
Supplement: fiaf016_Supplemental_Files [file fiaf016_supplemental_files.zip › Costa.Supplementary Material.docx]

**Supplementary Material**

**Suppl. Fig. S1**. Principal coordinate analysis (PCoA) plots of bacterial communities for sample type using Bray-Curtis dissimilarity matrix.

**Suppl. Fig. S2**. Principal coordinate analysis (PCoA) plots of single-celled eukaryotic communities for sample type using Bray-Curtis dissimilarity matrix.

**Suppl. Fig. S3.** Maximum likelihood phylogeny of the *Vibrio harveyi* clade estimated using nucleotide sequences of the 16S gene. Fish silhouettes represent individuals identified in this study. The scale bar represents the number of nucleotide substitutions per site. Tree was midpoint rooted for clarity only.

**Suppl. Table S1.** Composition of host sequence reads.

**Suppl. Table S2.** Description of vertebrate-associated viruses.

**Suppl. Table S3.** Host library information.
