# Supplementary figures and images for: Limited similarity in microbial composition among coral reef fishes from the Great Barrier Reef, Australia
Source: FEMS Microbiol Ecol. 2025 Feb 6;101(3):fiaf016. doi: 10.1093/femsec/fiaf016 (PMC11879539; doi:10.1093/femsec/fiaf016)

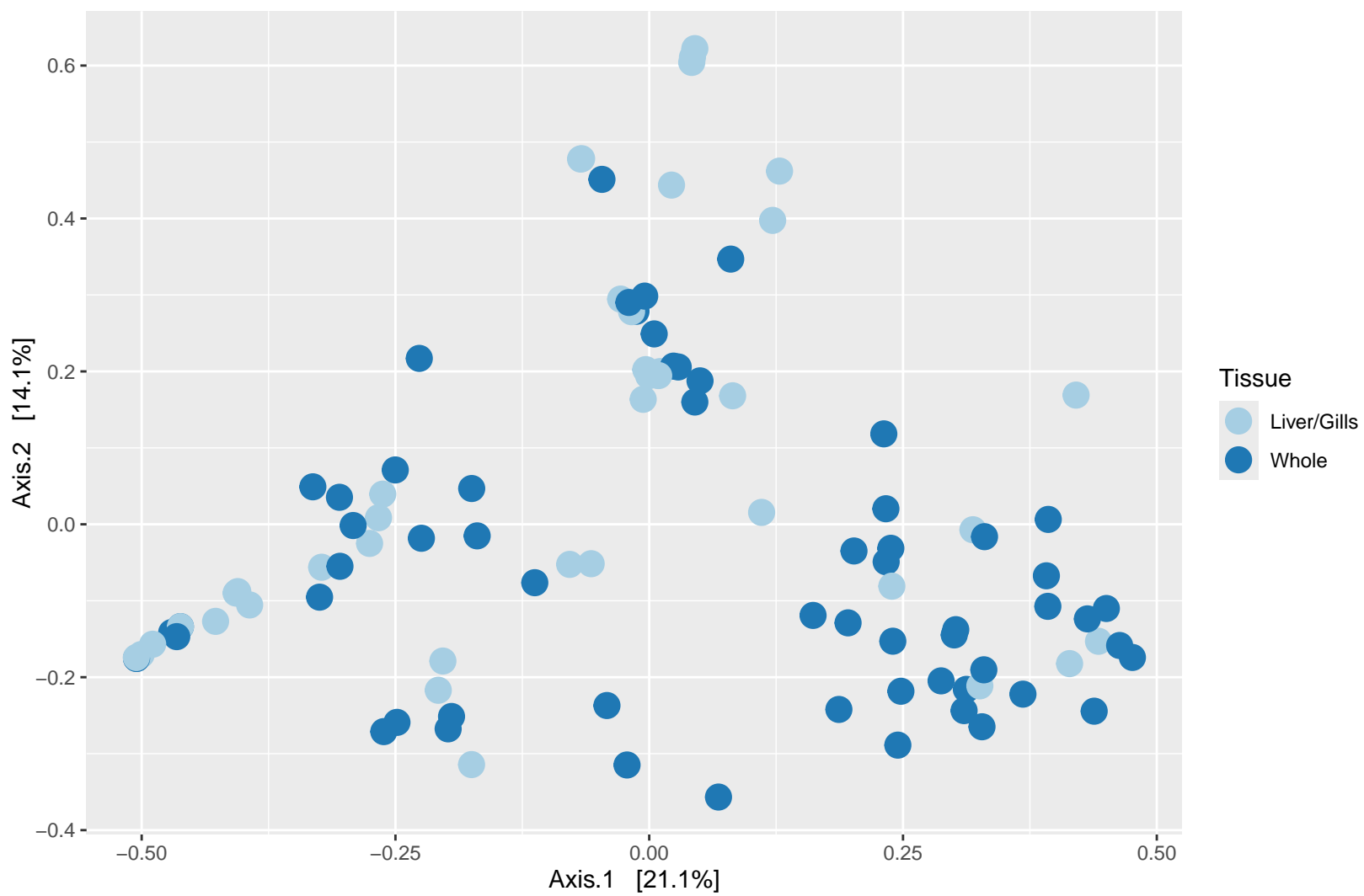

Supplement: fiaf016_Supplemental_Files [file fiaf016_supplemental_files.zip › Costa.Figure S1.pdf]

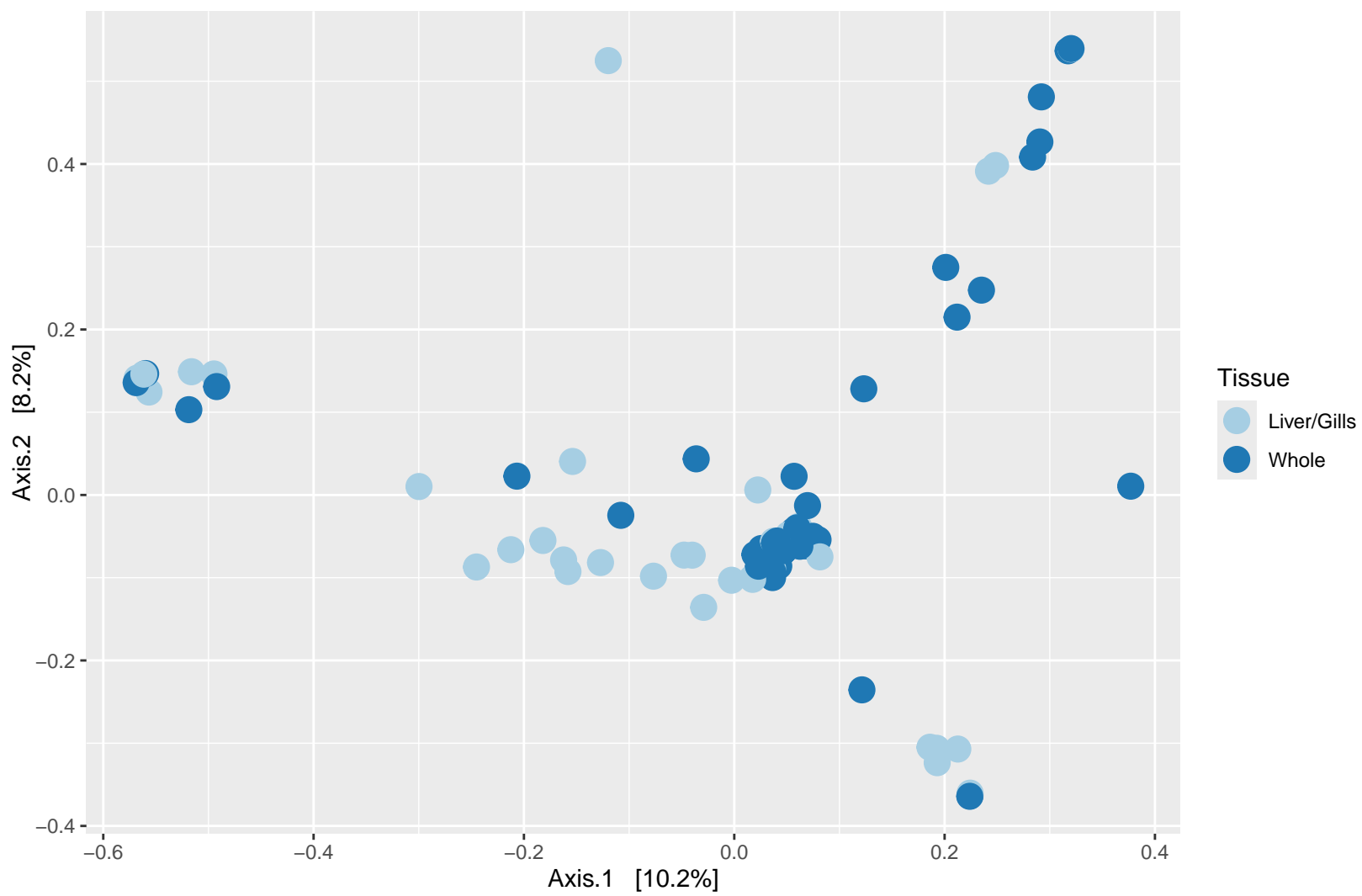

Supplement: fiaf016_Supplemental_Files [file fiaf016_supplemental_files.zip › Costa.Figure S2.pdf]

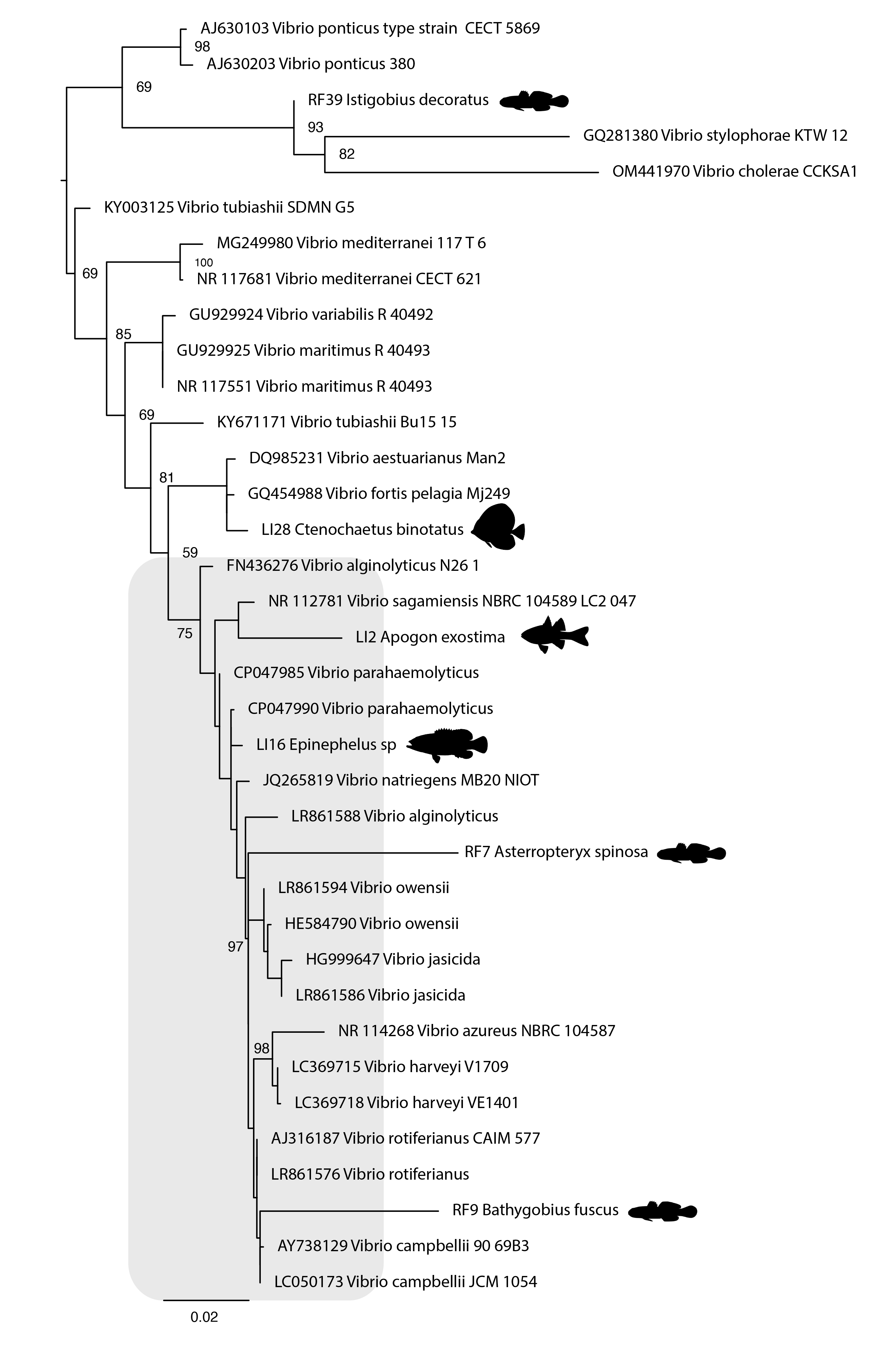

Supplement: fiaf016_Supplemental_Files [file fiaf016_supplemental_files.zip › Costa.Figure S3.tif]
